# 

**Published:** 1858-02

**Authors:** 


### CONTENTS OF THE FEBRUARY NUMBER.

### ORIGINAL COMMUNICATIONS.

Page

ART. I.—Reduction of Dislocations of the Hip-Joint by Manipulation. By F. H. Hamilton, M. D., 514

ART. II.—Fractures of the Sternum. By Frank H. Hamilton, M. D., - - - - - - 520

ART. III.—Fractures of the Inferior Maxilla. By O. C. Gibbs, M. D., - - - - - - - 526

ART. IV.—Reports of Cases occurring in the Medical Ward of the Buffalo Hospital. Reported by Dr. A. W Nichols, - - - - - - - - - - - - - - - - - 527

ART. V.—Alcoholic Treatment of Tuberculosis. By Thomas Cushing, - - - - - - 532

ART. VI.—Abstract of the Proceedings of the Buffalo Medical Association, - - - - - 536

ART. VII.—A Theoretical and Practical Treatise on Midwifery, - - - - - - - - 547

ART. VIII.—The Medical Student's Vade Mecum, - - - - - - - - - - - 551

ART. IX.—Monthly Record of Deaths in the City of Buffalo. By C. L. Dayton, M. D., Health Physician, - - - - - - - - - - - - - - - - - - 553

### ECLECTIC DEPARTMENT.

Microscopical Examination of Spots of Blood, - - - - - - - - - - - 555

### EDITORIAL DEPARTMENT.

Annual Dinner of the Erie Co. Medical Society, - - - - - - - - - - - 571

Reproduction of Bones and Joints after their removal in cases of Whitlow, - - - - - 572

Sorghum, or Chinese Sugar Cane, - - - - - - - - - - - - - - 583

